# Empathy and Violence in Schizophrenia and Antisocial Personality Disorder

**DOI:** 10.3390/healthcare12010089

**Published:** 2023-12-30

**Authors:** Konstantinos Tasios, Athanasios Douzenis, Rossetos Gournellis, Ioannis Michopoulos

**Affiliations:** 2nd Department of Psychiatry, Medical School, Attikon Hospital, National and Kapodistrian University of Athens, 12462 Haidari, Greece; thandouz@med.uoa.gr (A.D.); rgourn@med.uoa.gr (R.G.); imihopou@med.uoa.gr (I.M.)

**Keywords:** affective, antisocial, cognitive, empathy, personality, schizophrenia, theory of mind, violence

## Abstract

A paucity of cognitive and affective features of empathy can be correlated with violent behavior. We aimed to identify differences in empathy among four groups in a sample of 100 male participants: (1) 27 violent offenders with schizophrenia, (2) 23 nonviolent patients with schizophrenia, (3) 25 patients with antisocial personality disorder, and (4) 25 subjects from the general population, who formed the control group. Schizophrenia symptoms were quantified with the Positive and Negative Syndrome Scale. Empathy was measured with the empathy quotient. Theory of mind was evaluated using (a) the first-order false-belief task, (b) the hinting task, (c) the faux pas recognition test and (d) the “reading the mind in the eyes” test (revised). Differences noted among the groups were age (controls were younger) and educational status (antisocials were less educated). The empathy quotient scoring (*p* < 0.001) and theory-of-mind tests (*p* < 0.001) were distinct between the control group and the three other groups of participants, but not among the three patient groups. Patients with antisocial personality disorder, violent psychotic offenders and psychotic nonviolent patients show no remarkable differences in affective or cognitive empathy tests, but they all present deficits in empathy and theory of mind when compared to controls.

## 1. Introduction

Empathy is the ability to perceive the inner frame of reference of another person accurately, and with those emotional characteristics and meanings as if we were the other person, without losing the “as if” condition [1]. It allows understanding intentions, predicting behavior, and experiencing emotions triggered by each other’s emotions. It is a necessary component of normal social functioning [2]. Empathy allows building social connections with others, responding appropriately to social situations, forming and maintaining meaningful social bonds, and fostering healthy relationships.

Affective empathy refers to the ability to perceive, recognize, and experience another person’s emotions [3]. It is mainly assessed by emotion recognition tests, which depict basic emotions, such as joy, sadness, fear, anger, surprise, and disgust.

Deficits in emotion recognition tests are described in the first psychotic episode and schizophrenia [4] and persist despite antipsychotic treatment [5]. However, they do not seem to be specific to psychosis, as patients with psychopathic traits and personality disorders also have deficits, especially in the recognition of fear and sadness [6]. The inability to properly perceive, recognize and attribute emotions could lead to bias and perception of neutral situations as potentially hostile, with an increased likelihood of violent reaction.

The theory of mind (ToM) refers to the cognitive part of empathy and is defined as the ability to perceive and capture the mental state and emotions of another person. It allows anticipating and understanding the behavior of the other individual [2]. First-order theory of mind concerns the attribution of a thought to a person about the state of the world. Second-order theory of mind is more complex and involves the ability to perceive the thoughts of a second person for the thoughts of a third person about a particular situation or event [7].

Deficits in theory of mind have been repeatedly reported in schizophrenia, both in the first episode [8] and in its chronic form [9,10]. These could bring about a divergent tendency in the internal representation of others’ intentions, leading to beliefs that others are concealing their true intentions and eventually to the manifestation of delusions of persecution [11]. Similar symptoms and patterns of attribution of intent could lead to the use of violence [12,13]. Patients with schizophrenia and a history of violence appear to perform worse than patients with personality disorder on second-order ToM tests [14,15], a finding possibly related to the cognitive deficits found in schizophrenia [16]. There is also evidence that patients with schizophrenia and a history of violence perform better on second-order ToM tests versus patients with schizophrenia and no history of violence [17], possibly suggesting better cognitive performance.

Violent patients with personality disorder, primarily antisocial personality disorder, perform similarly to healthy controls on first- and second-order ToM tests [18]. Given that people with psychopathic characteristics do not have deficits in ToM [19], it is concluded that aggression in antisocial personality disorder with or without psychopathic characteristics does not appear to be associated with experimental or clinically established deficits in ToM. Therefore, the aim of this study was to investigate whether there are differences in measures of both cognitive and affective empathy in a sample of patients with schizophrenia, patients with schizophrenia who have committed a violent offence and patients with antisocial personality disorder. We formulated two hypotheses: (1) There are differences in empathy between patient groups and controls, and (2) Patients with antisocial personality disorder show less empathy than patients with schizophrenia with a history of a violent offence and the latter show less empathy than patients with schizophrenia and no history of violent offence.

## 2. Materials and Methods

### 2.1. Participants

The study was conducted between the years 2017 and 2022 and was approved by the Attikon University General Hospital Review Board (approval code ΕΒΔ1764/30-8-16) on 15 September 2016. A total sample of 100 male participants was consecutively recruited from: (a) the Forensic Psychiatry outpatient clinic of the 2nd Department of Psychiatry (Attikon Hospital), (b) the Korydallos Prison Psychiatric Clinic (Athens) and (c) the Agioi Anargyroi Health Centre COVID-19 Vaccination Department.

The sample was divided into four groups: (1) 27 patients with schizophrenia and history of committing a violent offence, (2) 23 patients with schizophrenia with no history of committing a violent offence, (3) 25 participants with antisocial personality disorder and (4) 25 general population participants comprising the control group. Sociodemographic and important clinical variables are presented in Table 1.

A violent offence was defined as homicide, assault, robbery, arson, any sexual offense (rape, sexual coercion, child molestation, indecent exposure, or sexual harassment), illegal threats, or intimidation. Lifetime occurrence was used as a temporal measure of violence.

Exclusion criteria for all participants included age under 18 years or over 65 years, any coexisting psychiatric, neurological or neoplasmatic disorder, and compromised ability to understand or read/write Greek. Patients with comorbidity of schizophrenia and antisocial personality disorder were excluded.

After a complete description of the study, every participant gave written informed consent. The study was approved by the local ethics committee.

### 2.2. Assessments

An experienced clinical psychiatrist performed the clinical evaluation of the participants using the DSM 5 diagnostic criteria.

Schizophrenia symptoms were quantified using the positive (P), negative (N) and general psychopathology (G) subscales of the Positive and Negative Syndrome Scale (PANSS) [20]. It has been translated and validated in Greek [21]. The patient is rated from 1 to 7 on 30 different symptoms based on the interview. The PANSS consists of three subscales, as follows.

The positive scale includes 7 items. The score ranges between a minimum of 7 and a maximum of 49. The items included are delusions, conceptual disorganization, hallucinations, excitement, grandiosity, suspiciousness/persecution and hostility.

The negative scale includes 7 items. The score ranges between a minimum of 7 and a maximum of 49. The items included are blunted affect, emotional withdrawal, poor rapport, passive/apathetic social withdrawal, difficulty in abstract thinking, lack of spontaneity and flow of conversation and stereotyped thinking.

The general psychopathology scale includes 16 items. The score ranges between a minimum of 16 and a maximum of 112. The included items are somatic concern, anxiety, guilt feelings, tension, mannerisms and posturing, depression, motor retardation, uncooperativeness, unusual thought content, disorientation, poor attention, lack of judgment and insight, disturbance of volition, poor impulse control, preoccupation and active social avoidance.

The total PANSS score ranges from a minimum of 30 to a maximum of 2101. As 1 rather than 0 is given as the lowest score for each item, a patient cannot score lower than 30 for the total PANSS score. Scores are often given separately for the positive items, negative items, and general psychopathology.

Theory of mind (ToM) was assessed using the following four tests in order of increasing difficulty.

(a) First-order false-belief task [9,22,23,24,25]. Two stories that are read aloud to the subject comprise this test, followed by two specific questions. The first question focuses on the mistaken belief that the story character (hero) has for the described situation. The knowledge of the mental state of that character is required for answering this question (ToM question) correctly. In order to corroborate that the subject understands the situation, a second question is posed concerning the situation, which does not require ToM skills to be answered correctly (reality question). For example, John has left 5 cigarettes in his pack. He places the package on the table and leaves the room. Meanwhile, Eugenia enters the room, picks up one of John’s cigarettes and leaves the room, without John’s knowledge. The ToM question is: “When John comes back for his cigarettes, how many cigarettes does he think he has left?” The reality question is: “How many cigarettes are really left in John’s pack?” The correct answer to the ToM question gets 1 and the wrong answer gets 0. In any case, the Tom question is followed by the reality question. It determines whether there is a problem in comprehension or memory function. If the examinee answers the reality question incorrectly, the correct answer to the ToM question is canceled (gets 0). The correct answer to the reality question after an incorrect answer to the ToM question does not change the score (i.e., 0).

(b) Hinting task [9,22,23,24,25]. This test examines the subject’s ability to deduce the real intentions hidden behind direct speech. Ten stories comprise the test with two participating characters in a social interaction, where one of the characters obviously hints at something. The participant is asked for the meaning of the hint. The correct answer is awarded two points. If not answered correctly, a more obvious innuendo is then provided and the correct answer is awarded one point. For example, Rebecca’s birthday is approaching. She tells her father, “I love animals, especially dogs!” The hinting task question is: “What does Rebecca really want to say when she says this?” An exact answer would be: Rebecca wants to say, “Dad, are you going to get me a dog for my birthday?” If the subject fails to answer or gives the wrong answer, a more obvious hint is given: Rebecca went on to say, “Will the pet shop be open on my birthday, Dad?” The new hinting task question is: “What would Rebecca want her dad to do?” A correct answer would be: Rebecca wants her dad to buy her a dog for her birthday or would like to get her a dog.

(c) Faux pas recognition test [24,25,26,27,28,29,30]. This test includes twenty stories, where ten of them contain a social faux pas. A faux pas is an embarrassing or awkward situation occurring when one of the story characters says something better left unsaid. Upon completion of the stories, participants were asked if they had a notion of a faux pas. If they had, the following questions were asked: (a) Who said it?; (b) Why shouldn’t it have been said?; (c) Why had it been said?; and (d) How did the other person in the story feel? The sum of correct detections of faux pas plus the sum of correct rejections (i.e., lack of a faux pas in a story) forms the faux pas—recognition score, which is regarded as a general measure of ToM capacity. The sum of correct responses that show that the participant grasped that the story character who was responsible for the faux pas either did not know something or did not realize something accounts for the faux pas—cognitive ToM score [31]. The sum of correct responses about how the “faux-pas victim” felt refers to the empathic ability score, which is considered a measure of emotional perspective taking [29,32].

The following story includes a faux pas: DN, director of an IT company, called a meeting of all the staff. “I have something to announce to you,” he said. “Mr. YA, one of our accountants, is very ill with cancer and is in hospital.” Everyone was stunned to hear the news, and at that very moment, P, a computer technician, arrived late. “Well, listen to what a sick joke I heard last night,” he said. “What did a patient with terminal cancer tell his doctor?” The questions posed to the participant are:

Did someone say something they shouldn’t have said or something inappropriate?

(A) If yes, who said something they shouldn’t have said or something inappropriate?

(B) Why shouldn’t he say it or why was it inappropriate?

(C) Why do you think he said it?

(D) When Peter arrived, did he know that the accountant had fallen ill with cancer?

(E) How do you think DN felt?

Recognition of faux pas (RFP) is the sum of 1. The score on the first question (Did someone say something they shouldn’t have said or something inappropriate?) for the faux pas stories (1 point for correct detection) and control stories (2 points for correct rejection) and 2. The score on the second question (Who said something they shouldn’t have said and or something inappropriate?) only for the faux pas stories (1 point for correct identification).

Understanding of faux pas (UFPA)—affective ToM is the sum of scoring on the third question (Why shouldn’t he say it or why was it inappropriate?) of the faux pas stories (1 point for a correct answer).

Understanding of Faux Pas—cognitive (UFPC) ToM is the sum of scoring on the fourth question (Why do you think he said it?) of the faux pas stories (1 point for a correct answer).

Empathic ability (EA) is the sum of scoring on the sixth question (How do you think X felt?) of the faux pas stories (1 point for a correct answer).

(d) Reading the mind in the eyes test (RME) (revised) [30,33]. This test evaluates the participant’s ability to recognize someone’s mental state by looking at a picture showing only a person’s eyes. Thirty-six separate black-and-white photographs of eyes are presented consecutively on a computer screen framed by four words representing possible complex mental states of the eye beholder. The participant chooses the best-describing word of the mental state reflected in the eyes of the particular photograph. Each correct answer scores one point. The participant is also asked to judge the gender of each person of each photograph as a control task. RMETOTAL is the sum of correct answers, while RMERATIO is the sum of correct answers provided the control questions are correct. The RME is considered an advanced ToM test focusing on measuring affective ToM, though assessing the cognitive state of a person as well (e.g., fantasizing, skeptical, decisive). The key difference from other advanced ToM tests is assessing the ability to decipher the emotions and thoughts from the depicted person’s eye area (mental state decoding) instead of deducing them from sentences, actions, or social circumstances (mental state reasoning). It is also regarded as a measure of cognitive empathy [34].

The false belief task, hinting task, faux pas test and reading the mind in the eyes test are not scales or questionnaires. We used the widely accepted translations from Cambridge University found at https://www.autismresearchcentre.com/tests/ (accessed on 1 July 2017) These translations have been used in the literature [24,25,28,29,30].

Empathy was evaluated using the empathy quotient (EQ). The EQ is a self-assessment tool used to measure empathy in normal-intelligence adults. It can be found at www.autismresearchcentre.com (accessed on 1 July 2017). It was conceived as a clinical tool used to detect lack of empathy in patients with psychopathology [2]. It has been validated in Greek [35]. The EQ consists of 60 distinct statements, divided into two types of sentences: 40 sentences regarding empathy and 20 filler sentences. The role of the 20 filler sentences was to distract the participant from the persistent focus on empathy. Responses are given on a 4-point Likert scale. Each of the sentences listed above scores 1 point if the participant supports the empathic behavior mildly and 2 points if they support it strongly. The affective and cognitive components are mixed. The final score ranges from 0 to 80 points. A sentence regarding empathy is: “I can sense if I am intruding, even if the other person doesn’t tell me”. The participant is asked to choose between “strongly agree”, “mildly agree”, “mildly disagree”, and “strongly disagree”. The points awarded for each choice are 2, 1, 0, and 0, respectively. A filler sentence is: “I think that good manners are the most important thing a parent can teach their child.” The participant is asked to choose between “strongly agree”, “mildly agree”, “mildly disagree”, and “strongly disagree”. Each answer is awarded 0 points.

Cronbach’s α was calculated in order to test for internal consistency of the tools used. Values were: false belief task 0.78, hinting task 0.71, empathy quotient 0.82, and reading the mind in the eyes 0.57.

### 2.3. Statistical Analysis

The Pearson χ^2^ test for comparison of percentages, one-way ANOVA (with Bonferroni post hoc comparisons) for comparison of means of variables presenting normal distribution, and Kruskal–Wallis nonparametric analysis of variance for variables that were not normally distributed were used for the statistical analysis of the data. Correlations were tested by the Spearman rho coefficient, since most of the variables were not normally distributed. The internal consistency of the scales was calculated with Cronbach’s alpha coefficient. Bonferroni correction for multiple comparisons was used and the statistical significance level was set to *p* = 0.05/5 = 0.01. Statistical analysis was carried out using SPSS (Version 24.0) for Windows.

## 3. Results

Demographic and clinical characteristics of the sample are presented in Table 1. The four groups differed in age, as controls were younger, and education, as antisocial patients were less educated. There were no significant differences in duration of illness, age of illness onset, number of relapses and admissions. Differences in PANSS scoring were not significant between antisocial, schizophrenic and violent schizophrenic patients, while healthy controls scored significantly lower than the patient groups in all three PANSSs.

As shown in Table 2, every patient group scored lower than healthy controls in EQ, with no significant difference in EQ scoring between groups. The three patient groups also scored significantly lower than controls in the false-belief task, hinting task and every faux pas test, while differences between them were not significant. Healthy controls scored higher than all patient groups in reading the mind in the eyes, with differences between them being nonsignificant.

As demonstrated in Table S1 (Supplementary Materials), correlation analysis showed a negative correlation between PANSS scores and every cognitive and emotional empathy measure, with the exception of the false-belief task, where correlation was not significant. There was also a positive correlation among cognitive empathy variables as well as between cognitive and emotional empathy variables. Finally, age was positively associated with PANSS general psychopathology score and negatively with faux pas recognition, faux pas understanding and reading the mind in the eyes scores.

## 4. Discussion

This study aimed to identify variations in empathy measures between patients with schizophrenia, schizophrenia and a history of violence and antisocial personality disorder. The data obtained indicated significant differences between every patient group and controls in both cognitive and emotional aspects of empathy, while no significant differences were found among patient groups.

Previous research has focused on facial affect recognition tasks as measures of affective empathy, indicating a clear deficit across most emotions in patients with schizophrenia and a history of violence when compared to healthy controls [36], but also producing mixed results when comparing them with nonviolent patients. Violent patients showed a significantly better ability to identify facial emotional expressions but a poorer ability to discriminate between intensity of emotions than nonviolent schizophrenia patients [37] or attributing dominance to faces [38]. These studies argue that patients with schizophrenia and a history of violence show diminished empathic ability, but still better than nonviolent patients with schizophrenia. Inability to correctly identify facial emotional cues could lead to a lack of comprehension of social scenarios or the motives of other individuals. This might also hinder the recognition of signs of distress in others, consequently eliminating the usual restraint that these signals tend to provoke against violent behavior.

Studies on theory of mind also elicit contradictory results. Patients with schizophrenia and a history of violence seem to display deficits compared to those with antisocial personality disorder or healthy controls [39]. In addition, it has been argued that violent schizophrenic patients have poorer second-order ToM [14], are less able to interpret emotion information from the eyes, and have poorer performance on ToM tasks [15] compared to a personality disorder group, suggesting that cognitive empathy is more disrupted in schizophrenia than in antisocial personality disorder.

On the contrary, a study showed that violent schizophrenic patients performed better at second-order ToM tasks and the cognitive component of faux pas tasks, while performing worse at recognizing faux pas and empathic inference tasks when compared with schizophrenic patients [17]. Moreover, the ability to infer a cognitive state in others and the ability to recognize faux pas significantly predicted the likelihood of a history of violence. This controversial finding suggested that patients who performed better in certain cognitive empathy tests were more likely to have inflicted violence in the past. Conversely, when comparing forensic with non-forensic patients with schizophrenia, no significant differences were found in ToM, while both groups performed worse than controls [40]. Also, comparing psychotic patients with controls demonstrated statistically significantly lower scores for the group of subjects with a diagnosis of psychosis on both the hinting task and reading the eyes in the mind test [41]. Contrarily, subjects with antisocial personality disorder performed worse than controls on the reading the mind in the eyes test, indicating that patients diagnosed with psychosis have deficits in social reasoning and emotion recognition tasks, while patients with antisocial personality disorder showed lower scores only in emotion recognition tests. The conclusion from the sum of these studies is that patients with schizophrenia with or without a history of violence have deteriorated empathic ability, as displayed in cognitive and affective empathy measures, although no robust differences can be found between those two categories of psychotic patients.

The results of our study showed differences between violent schizophrenic patients and controls on the reading the eyes in the mind test, the hinting task, and EQ, with controls scoring significantly higher. These data confirm that violent schizophrenic patients have a clear deficit in empathy tasks, both cognitive and emotional, although no significant differences were observed when they were compared with patients with schizophrenia or antisocial personality disorder. Corroborating previous studies, our data confirmed that both violent and nonviolent patients with schizophrenia have disturbed empathic abilities, displayed across a variety of tests, that could enhance the risk of violent behavior. Difficulties in generating prosocial behavior and misinterpretation of social cues may perpetuate offending and reoffending and hinder psychological therapeutic interventions.

Facial affect recognition studies on antisocial personality disorder have produced contradictory results. Non-psychopathic antisocials showed impairments in the recognition of basic emotions compared with controls and psychopathic antisocials, while no significant group differences were found for complex emotions [18]. When looking specifically at reading emotion from the eyes, non-psychopathic antisocials were more impaired at recognizing basic emotions than healthy controls. In another study, the antisocial personality disorder group performed worse in recognizing sad facial affect [42]. In the same group, those with high scores on psychopathy were less accurate than low scorers at classifying sad facial affect. There was also a significant negative correlation between total psychopathy score and sad affect recognition accuracy. An older meta-analysis focusing on affect perception in psychopathy detected deviations in recognizing happiness, sadness, fear and surprise [43]. A clear conclusion can be made that patients with antisocial personality disorder, both psychopathic and not, show limited ability to empathize, although differences between them cannot be robustly identified.

ToM deficits are evident in antisocial personality disorder, especially in attributing a mental state and empathic understanding in faux pas scenarios while preserving the ability to recognize a faux pas [18]. The same study showed no significant differences between antisocials and controls on first- and second-order ToM or faux pas, while another study found that antisocial personality disorder patients had poorer performance on second-order ToM [31].

Similarly, a study comparing individuals with a history of serious assault with controls showed that aggressive participants displayed reduced empathic responses to a social video task that differentiates empathy and ToM, while no ToM deficits were found [44].

Regarding types of antisociality, individuals in the psychopathic type fail to implicitly engage in cognitive empathy when explicitly required. Individuals in the antisocial-only subtype appear able to engage in cognitive empathy, showing no differences on questionnaire or behavioral tasks that tap explicit cognitive empathy, but may display subtle difficulties accurately inferring the emotions of others [45]. It has been suggested that while psychopathy may generally interfere with metacognitive abilities (including attributing a mental state to others), individuals with high levels of psychopathy do not exhibit this deficit. It is as if the impact of psychopathy on emotion processing is not linear, but rather plateaus at a certain point [46].

In our study, patients with antisocial personality disorder performed significantly worse than controls on ToM and emotional empathy tasks, underscoring a general deficit in empathy, while no significant differences were found between them and the other patient groups. A lack of understanding of others’ emotions and opinions may contribute to lack of guilt for mistreating others and further reinforce antisocial behaviors.

Moreover, a negative correlation was found between PANSS scores and empathy and theory of mind scores. This finding suggests that as psychopathological features accumulate during the course of schizophrenia, empathic ability tends to wane. A positive correlation was also found between cognitive and emotional empathy variables, suggesting that cognitive and emotional empathy are complementary concepts. Another positive correlation was found between age and PANSS general psychopathology, while age correlated negatively with faux pas recognition, faux pas understanding and reading the mind in the eyes scores. This finding may signify that the elderly are more susceptible to mental strain and may gradually lose the ability to empathize as they get older.

Empathy studies can contribute to risk assessment in forensic psychiatry. For instance, lack of empathy is often associated with antisocial behavior and may be used as an indicator of risk of violent or criminal behavior. Moreover, understanding a patient’s capacity for empathy can inform individualized treatment plans. Treatments such as cognitive behavioral therapy (CBT) can be tailored to help individuals improve their empathic abilities. Also, in court cases, understanding the role of empathy in a defendant’s mental state can have significant legal implications. It can influence decisions about culpability, sentencing, and the potential for rehabilitation. Forensic psychiatry often involves a delicate balance between serving the justice system and maintaining the principles of beneficence and non-maleficence, which are central to clinical medical ethics. The use of empathy in examination and evaluation is a topic central to this ongoing discussion [47].

Limitations of our study include the small sample, although similar to other studies, and lack of matching for age, educational and intelligence level. Cross-sectional design and the use of self-reported questionnaires may affect the generalizability of its results. Research findings are not always easily generalized to everyday life. Still, our study provides initial data and identifies correlations that can then be further investigated in longitudinal studies. The main strength of our study is that it is the first time these groups have been compared head to head.

## 5. Conclusions

Schizophrenia patients with a history of violence and those with no history of violence had no significant differences in illness duration, age of illness onset, number of relapses or admissions. Educational level was lower in patients with antisocial personality disorder, and controls were younger. Differences in PANSS scoring were not statistically significant between the three patient groups, while controls scored significantly lower. This finding could suggest that psychotic symptoms may be a psychopathological feature of patients with antisocial personality disorder, especially those incarcerated or followed up in a forensic psychiatry department.

Differences in empathy, as measured with the EQ, were not significant among the three patients’ groups either. Patients with schizophrenia, either violent or nonviolent, as well as patients with antisocial personality disorder scored significantly lower than controls. An empathy deficit is obvious in all three patient groups, and no distinctive differences can be applied to any of the patient groups.

Group comparison according to theory of mind produced similar results. While differences between controls and the three patient groups were significant, with controls scoring higher in the hinting task, faux pas test and reading the mind in the eyes test, no differences were found among patient groups, underlying a common deficit in theory-of-mind capacity in schizophrenia and antisocial personality disorder.

Research in forensic psychiatry focusing on reoffending risk assessment after incarceration is crucial when deciding on the treatment and management of forensic psychiatry patients. Results from this research can yield tools that can be incorporated into clinical practice calculating the probability of reoffending, thus contributing to public safety by preventing the premature release of possible reoffenders. Studies with larger participant numbers need to be conducted, incorporating samples of patients with or without psychotic disorders that are prone to violence or have been incarcerated for committing a violent crime. Empathy assessment is a highly promising field of research among forensic scientists and should be pursued with rigor, especially in academic institutions.

## Figures and Tables

**Table 1:** Demographic and clinical characteristics of patients with antisocial personality disorder (AS), schizophrenia (S), schizophrenia with a history of violence (SV) and healthy controls (HCs).

	AS Mean (SD)		S Mean (SD)		SV Mean (SD)		HC Mean (SD)		*p*
Age (years)	44.32	11.99	40.96	9.65	43.26	9.88	33.46	10.62	0.002
Education (years)	8.83	3.62	13.10	2.71	11.92	3.16	11.21	2.70	<0.001
Living alone	15		8		14		5		0.022
Duration of illness (years)	-	-	11.82	7.78	13.24	8.03	-	-	0.539
Age of onset (years)	-	-	29.30	8.51	31.04	10.00	-	-	0.522
Relapses	-	-	3.89	5.34	2.28	1.40	-	-	0.225
Admissions	1.13	0.83	3.00	3.91	2.33	1.55	-	-	0.106
PANSSPTOTAL	18.95	6.68	16.00	6.12	19.91	8.59	7.04	0.20	<0.001
PANSSNTOTAL	19.73	6.79	27.00	7.31	27.86	5.18	7.00	0.00	<0.001
PANSSGTOTAL	38.81	10.21	37.09	11.25	40.19	11.90	16.28	0.98	<0.001

PANSS = Positive and Negative Syndrome Scale, PANSSPTOTAL = positive symptoms score, PANSSNTOTAL = negative symptoms score, PANSSGTOTAL = general psychopathology score (all comparisons by one-way ANOVA, except for PANSS (Kruskal–Wallis)).

**Table 2:** Empathy and social cognition test performance of patients with antisocial personality disorder (AS), schizophrenia (S), schizophrenia with a history of violence (SV) and healthy controls (HCs).

	AS Mean (SD)		S Mean (SD)		SV Mean (SD)		HC Mean (SD)		*p*
EQTOTAL	29.09	5.22	30.96	6.33	31.12	9.69	43.40	9.74	<0.001
FBTOTAL	1.70	0.50	1.74	0.60	1.68	0.60	2.00	0	0.11
HTTOTAL	17.52	1.997	17.83	1.92	17.64	2.36	19.96	0.20	<0.001
RFP	31.86	4.97	29.91	4.60	31.24	6.70	36.90	0.81	<0.001
UFPA	6.48	2.54	6.13	2.16	6.19	2.44	10.00	0.00	<0.001
UFPC	3.67	2.87	4.57	2.46	3.71	3.15	9.08	1.04	<0.001
EA	6.43	2.59	6.04	2.27	6.14	2.29	10.00	0.00	<0.001
RMETOTAL	18.74	5.01	19.19	3.84	19.52	4.52	24.96	2.67	<0.001
RMERATIO	18.13	4.83	18.76	4.01	18.72	4.47	24.68	2.95	<0.001

EQ = empathy quotient, FB = false-belief task, HT = hinting task, RFP = recognition of faux pas, UFPA = understanding of faux pas (affective), UFPC = understanding of faux pas (cognitive), EA = empathic ability, RME = reading the mind in the eyes (comparison by Kruskal–Wallis test).

## Data Availability

Data may be obtained from the corresponding author.

## Supplementary Materials

The following supporting information can be downloaded at: https://www.mdpi.com/article/10.3390/healthcare12010089/s1, Table S1. Correlation analysis.
