# Supplementary material for: Empathy and Violence in Schizophrenia and Antisocial Personality Disorder
Source: Healthcare (Basel). 2023 Dec 30;12(1):89. doi: 10.3390/healthcare12010089 (PMC10779118; doi:10.3390/healthcare12010089)
Supplement: Supplementary file 1 [file healthcare-12-00089-s001.zip › healthcare-2769142-supplementary.pdf]

Table S1. Correlations

		Age (years)	Education (years)	PANSSP TOTAL	PANSSN TOTAL	PANSSG TOTAL	FBTTOTAL	HTTOTAL	RFP	UFPA	UFPAC	EA	RMETOTAL	RMERATIO	EQTOTAL
Age	r(p)	1.000	-0.038 (0.7)	0,291 (0.005)	0.256 (0.014)	0.353 (0.001)	-0.161 (0.119)	-0.022 (0.830)	-0.303 (0.004)	-0.375 (<0.001)	-0.333 (0.001)	-0.290 (0.006)	-0.351 (0.001)	-0.376 (<0.001)	-0.208 (0.043)
Education (years)	r(p)	-0.038 (0.717)	1	-0.151 (0,162)	0.089 (0.410)	-0.127 (0.244)	0.075 (0.483)	0.077 (0.476)	-0.039 (0.729)	-0.014 (0.900)	0.005 (0.963)	-0.040 (0.722)	0.252 (0.018)	0.235 (0.028)	0.168 (0.115)
PANSSPTOTAL	r(p)	0.291 (0.005)	-0.151 (0.162)	1	0.677 (<0.001)	0.858 (<0.001)	--0.185 (0.079)	-4.27 (<0.001)	-5.48 (<0.001)	-0.552 (<0.001)	-0.514 (<0.001)	-0.566 (<0.001)	-0.393 (<0.001)	-0.437 (<0.001)	-0.495 (<0.001)
PANSSNTOTAL	r(p)	0.256 (0.014)	0.089 (0.410)	0.677 (<0.001)	1	0.769 (<0.001)	-0.188 (0.074)	-0.597 (<0.001)	-0.641 (<0.001)	-0.694 (<0.001)	-0.581 (<0.001)	-0.652 (<0.001)	-0.450 (<0.001)	-0.463 (<0.001)	-0.400 (<0.001)
PANSSGTOTAL	r(p)	0.353 (0.001)	-0.127 (0.244)	0.858 (<0.001)	0.769 (<0.001)	1	-0.215 (0.042)	-0.499 (<0.001)	0.677 (<0.001)	-0.687 (<0.001)	-0.629 (<0.001)	-0.670 (<0.001)	-0.453 (<0.001)	-0.471 (<0.001)	-0.502 (<0.001)
FBTTOTAL	r(p)	-0.161 (0.119)	0.075 (0.483)	-0.185 (0.079)	-0.188 (0.074)	-0.215 (0.042)	1	0.195 (0.057)	0.210 (0.047)	0.262 (0.013)	0.128 (0.228)	0.267 (0.011)	0.120 (0.253)	0.135 (0.198)	0.255 (0.013)
HTTOTAL	r(p)	-0.022 (0.830)	0.077 (0.476)	-0.427 (<0.001)	-0.597 (<0.001)	-0.499 (<0.001)	0.195 (0.057)	1	0.575 (<0.001)	0.631 (0.001)	0.564 (<0.001)	0.659 (<0.001)	0.467 (<0.001)	0.456 (<0.001)	0.483 (<0.001)
RFP	r(p)	-0.303 (0.004)	-0.039 (0.729)	-0.548 (<0.001)	-0.641 (<0.001)	-0.677 (<0.001)	0.210 (0.047)	0.575 (<0.001)	1	0.866 (<0.001)	0.711 (<0.001)	0.842 (<0.001)	0.568 (<0.001)	0.569 (<0.001)	0.470 (<0.001)
UFPA	r(p)	-0.375 (<0.001)	-0.014 0.900	-0.0552 (<0.001)	-0.694 (<0.001)	-0.687 (<0.001)	0.262 (0.013)	0.631 (<0.001)	0.866 (<0.001)	1	0.835 (<0.001)	0.910 (<0.001)	0.638 (<0.001)	0.626 (<0.001)	0.491 (<0.001)
UFPC	r(p)	-0.333 (0.001)	0.005 (0.963)	-0.514 (<0.001)	-0.581 (<0.001)	-0.629 (<0.001)	0.128 (0.228)	0.564 (<0.001)	0.711 (<0.001)	0.835 (<0.001)	1	0.764 (<0.001)	0.446 (<0.001)	0.446 (<0.001)	0.474 (<0.001)
EA	r(p)	-0.290 (0.006)	-0.040 (0.722)	-0.566 (<0.001)	-0.652 (<0.001)	-0.670 (<0.001)	0.267 (0.011)	0.659 (<0.001)	0.842 (<0.001)	0.910 (<0.001)	0.764 (<0.001)	1	0.614 (<0.001)	0.600 (<0.001)	0.561 (<0.001)
RMETOTAL	r(p)	-0.351 (0.001)	0.252 (0.018)	-0.393 (<0.001)	-0.450 (<0.001)	-0.453 (<0.001)	0.120 (0.253)	0.467 (<0.001)	0.568 (<0.001)	0.638 (<0.001)	0.446 (<0.001)	0.614 (<0.001)	1	0.985 (<0.001)	0.428 (<0.001)
RMERATIO	r(p)	-0.376 (<0.001)	0.235 (0.028)	-0.437 (<0.001)	-0.463 (<0.001)	-0.471 (<0.001)	0.135 (0.198)	0.456 (<0.001)	0.569 (<0.001)	0.626 (<0.001)	0.446 (<0.001)	0.600 (<0.001)	0.985 (<0.001)	1	0.436 (<0.001)
EQTOTAL	r(p)	-0.208 (0.043)	0.168 (0.115)	-0.495 (<0.001)	-0.400 (<0.001)	-0.502 (<0.001)	0.255 (0.013)	0.483 (<0.001)	0.470 (<0.001)	0.491 (<0.001)	0.474 (<0.001)	0.561 (<0.001)	0.428 (<0.001)	0.436 (<0.001)	1

r=correlation coefficient, p=p value, PANSS=Positive and Negative Syndrome Scale, PANSSPTOTAL=Positive Symptoms score, PANSSNTOTAL=Negative Symptoms score, PANSSGTOTAL= General Psychopathology score, EQ=Empathy EQ=Empathy Quotient, FB=False Belief Task, HT=Hinting Task, RFP=Recognition of Faux Pas, UFPA=Understanding of Faux Pas(affective), UFPC=Understanding of Faux Pas(cognitive), EA=Empathetic Ability, RME=Reading the Mind in the Eyes.
